# Astragaloside IV inhibits ventricular remodeling and improves fatty acid utilization in rats with chronic heart failure

**DOI:** 10.1042/BSR20171036

**Published:** 2018-05-22

**Authors:** Bin Tang, Jin-Guo Zhang, Hong-Yong Tan, Xi-Qing Wei

**Affiliations:** 1 Department of Cardiology, Affiliated Hospital of Jining Medical University, Jining, Shandong 272000, China; 2 Department of Medicine, Shandong University Medical College, Jinan, Shandong 250100, China

**Keywords:** Astragaloside IV, chronic heart failure, energy metabolism, free fatty acid, rat, ventricular remodeling

## Abstract

Chronic heart failure (CHF) is the end-stage of many cardiovascular diseases and severely affects the patients’ lifespan. Inhibiting ventricular remodeling is thus a primary treatment target for CHF patients. Astragaloside IV (AS-IV) can improve cardiac function and protect myocardial cells. The study aims to investigate the effects of AS-IV on ventricular remodeling and explore its role in regulating energy metabolism using a rat CHF model. Sprague–Dawley rats were divided into five groups (*n*=20 per group): CHF + benazepril hydrochloride (Benazepril HCL), CHF + low-dose (30 mg.kg^−1^.day^−1^) AS-IV, CHF + high-dose (60 mg.kg^−1^.day^−1^) AS-IV, and a sham control group. After 8 weeks of treatment, the cardiac structure and functional parameters were measured. Morphological changes in the myocardial tissue in five groups were evaluated. Protein and mRNA expression of peroxisome proliferator-activated receptor α (PPARα), medium-chain acyl-CoA dehydrogenase (MCAD), and muscle carnitine palmitoyl transferase-1 (MCPT1) were also analyzed. Our results showed that the left ventricular mass index (LVMI), collagen volume fraction (CVF), and free fatty acid (FFA) concentration of CHF group rats increased when compared with sham control group, while the protein and mRNA expressions of PPARα, MCAD, and MCPT1 decreased in CHF. Importantly, treatment with AS-IV (CHF + AS-IV group) showed improved heart function and structure, increased expression of PPARα, MCAD, and MCPT1 and improved FFA utilization in comparison with CHF group. In conclusion, our study shows that AS-IV inhibits ventricular remodeling, improves cardiac function, and decreases FFA concentration of CHF model rats. Our findings suggest a therapeutic potential of using AS-IV in CHF.

## Introduction

Chronic heart failure (CHF), which severely affects the patients’ lifespan and quality of life, is the end-stage of many cardiovascular diseases and is a major public health problem [1–3]. According to epidemiological data, there are more than 5.8 million CHF patients in America and over 23 million CHF patients worldwide [4,5]. A 5- and 10-year survival rate of CHF patients is estimated to be 50 and 10%, respectively, and is even lower in developing countries [6,7]. Ventricular remodeling, including ventricular wall thickening, ventricle dilation, and collagen fiber proliferation, is an important pathological feature of CHF and contributes to ventricular dysfunction, and increase in oxygen consumption, and even cardiac death [8,9]. Inhibiting ventricular remodeling is thus a primary treatment target for CHF patients and a large number of studies have been carried out to achieve this objective [10].

Astragaloside IV (AS-IV) is an extract from the traditional Chinese medicinal plant *Astragalus* and has many pharmacological functions. AS-IV improves cardiac function, protects myocardial cells, amongst its other cardiovascular protective effects [11]. Many drugs containing AS-IV have been widely used in clinics for the treatment of cardiovascular diseases including coronary heart disease and heart failure, but the mechanism of its action remains unclear. Some studies have suggested that the protective effects of AS-IV on cardiac hypertrophy are associated with the regulation of cardiac-related signaling pathways [12–14]. In addition, AS-IV has been shown to reduce the number of apoptotic myocytes in rats [15]. AS-IV protects against ischemia and ischemia/reperfusion injury by initiating energy metabolism [16]. AS-IV also attenuates lipopolysaccharide-induced cardiac dysfunction by down-regulating inflammatory signaling in mice [17].

In the failing heart, cardiac function impairment is associated with alterations in energy substrate metabolism [18]. It has been previously elucidated that energy substrate of the hypertrophic myocardial cell shifts from free fatty acid (FFA) to glucose due to up- or down-regulation of mRNA and enzymes involved in energy substrate metabolism [19,20]. The key enzymes involved in FFA oxidation are medium-chain acyl-CoA dehydrogenase (MCAD) and muscle carnitine palmitoyl transferase-1 (MCPT-1), and expression of these enzymes is controlled by nuclear factor peroxisome proliferator-activated receptor α (PPARα) [21]. Thus, improving energy metabolism to treat heart failure may be a potential new treatment approach.

The present study was designed to examine the effects of AS-IV on CHF rats by assessing cardiac function and structure, hemodynamics and myocardial tissue morphology, and by exploring the mechanisms from an energy metabolism aspect. Considering that Benazepril hydrochloride (Benazepril HCL) is a type of angiotensin-converting enzyme inhibitor (ACEI) usually used to decrease cardiac after-load and inhibit ventricular remodeling [22–24], we incorporated a ‘Benazepril HCL group’ as a standard control group to compare the effect of AS-IV. We hypothesized that the cardiovascular protective effects of AS-IV may be due to the up-regulation of gene and enzyme expression related to FFA metabolism to improve energy metabolism in myocardial cells.

## Materials and methods

### Antibodies and reagents

AS-IV was purchased from Nanjing Spring and Autumn Biological Engineering (Nanjing, China). Benazepril HCL was purchased from Novartis Pharmaceuticals Corporation (Beijing, China). Polyclonal antibodies against PPARα, MCAD, glyceraldehyde phosphate dehydrogenase (GAPDH), and MCPT-1, and secondary antibodies were purchased from Boster Biological Engineering (Wuhan, China). Total RNA extraction kit and the cDNA synthesis kit were purchased from TIANGEN Biotechnology (Beijing, China). The primers for PPARα, MCAD, GAPDH, and MCPT-1 were designed and synthesized by Sangon Biotechnology (Shanghai, China). The Masson’s trichrome staining kit was purchased from Nanjing SenBeiJia Biological Technology (Nanjing, China). The FFA detection kit was purchased from ShangHai Chaoyan Biotechnology (Shanghai, China). The EP50 tube was purchased from BDTar BioTech (Shanghai, China).

### Animals and treatment

One-hundred male Sprague–Dawley rats (Experimental Animal Centre, Shanxi Medical University, Jinzhong, China; certificate number: SCXJ20150001) of SPF grade weighing 240 ± 10 g, were housed in a controlled environment (23 ± 2°C, 45 ± 5% humidity, and a 12-h dark/12-h light cycle). The experiments were conducted according to the principles approved by the Animal Care and Use Committee of Affiliated Hospital of Jining Medical University.

### CHF model establishment

Rats were divided into five groups (*n*=20 per group) in accordance with the random number table. The groups included a CHF group, a CHF + Benazepril HCL group, CHF + low-dose AS-IV, CHF+ high-dose groups, and a control sham operation group. CHF was induced by an abdominal aortic constriction (AAC) as described previously after assessing the baseline situation [25]. AAC was established using a 7-0 suture tied around the abdominal aorta in which a 20-gauge needle was inserted. The needle was then retracted yielding a 70% constriction with an outer aortic diameter of approximately 0.9 mm. In the sham operation group, the same surgery was performed as described above except that the aorta was not constricted. All animals had free access to water and a standard rodent chow.

### Evaluation of CHF model rats and drug treatment

The results of the model establishment were evaluated by ultrasonic cardiogram (UCG) after 6 weeks of the surgery. Serum FFA concentration was measured in all groups. All animals received drug/placebo treatment for 8 consecutive weeks after we confirmed that the CHF model was successfully established. The rats in CHF + low-dose and high-dose AS-IV groups were administered AS-IV through an intragastric gavage at 30 and 60 mg.kg^−1^.day^−1^ dissolved in 1 ml of 1% sodium carboxymethyl cellulose (CMC), respectively. Rats in the CHF + Benazepril HCL group were administered the drug through an intragastric gavage at 10 mg. kg^−1^.day^−1^ dissolved in 1 ml CMC. Rats in the sham operation and CHF group were administered CMC at 1 ml/day through an intragastric gavage as a placebo control.

### Post-treatment examination of cardiac structure and function

After 8 weeks of treatment, all five groups were anesthetized with 3% isoflurane, and the cardiac structural and functional parameters, including the left ventricular end-diastolic dimension (LVEDD), the left ventricular end-systolic dimension (LVESD), the left ventricular posterior wall depth (LVPWD), the left ventricular ejection fraction (LVEF), the left ventricular fractional shortening (LVFS), and the isovolumic relaxation time (IVRT) were determined noninvasively by echocardiography imaging using a Vevo 2100 high-resolution imaging system equipped with a transducer with center frequencies ranging from 13 to 24 MHz (MS250; Visual Sonics, Toronto, Canada).

### Evaluation of cardiac hemodynamic parameters

After conducting echocardiography, all groups underwent left ventricular intubation to confirm cardiac function examination. Rats were anesthetized with 20% urethane (0.5 ml/100 g, i.p.) and fixed on an operation table, and the EP50 tube, connected with a pressure transducer, was inserted into the left ventricle from the right common carotid artery. After connecting the pressure sensor with the physiological signals collection system, the hemodynamic parameters, including the left ventricular end-diastolic pressure (LVEDP), the left ventricular end-systolic pressure (LVESP) and the maximal rate of the increase/decrease in the left ventricular pressure (±d*p*/d*t*) were monitored.

### Detection of serum and myocardium FFA concentration and ventricular remodeling parameters

After left ventricular intubation, rats were killed by an intraperitoneal injection of sodium pentobarbital (20 mg/kg), and blood samples were collected to detect the FFA concentration. Hearts were removed and the weight of the left ventricle and whole heart were measured with an automatic analytical balance to calculate the left ventricular mass index (LVMI) (the ratio of the left ventricular weight to the body weight) and the heart mass index (HMI) (the ratio of the heart weight to the body weight). Part of the left ventricle was homogenized to detect the FFA concentration in the myocardium, and part of left ventricle was preserved in a −80°C freezer for Western blot and real-time quantitative PCR (RT-qPCR) analyses. Remaining left ventricle was fixed in formalin for histological examinations.

### Histological examinations

After fixation for 72 h, the myocardial tissue was dehydrated and embedded in paraffin and samples were sliced into pathological sections (4 μm). Pathological sections from the same rat were stained with Hematoxylin-Eosin (H&E, Nanjing Jiancheng Bioengineering Institute, Nanjing, China) and Masson’s trichrome. The H&E stained sections were observed under a light microscope (Olympus Medical Systems Corp, Tokyo, Japan), and the myocyte cross-sectional area of different groups was measured to confirm whether there was a difference amongst five experimental groups in the myocardial morphology. Sections were stained with a Masson’s trichrome kit. We calculated collagen volume fraction (CVF) using image analysis system IPP 6.0, and compared the differences amongst groups.

### Western blot analysis

Heart tissue was lysed in RIPA buffer containing a mixture of protease inhibitors (Santa Cruz Biotechnology, Texas, U.S.A.) to extract proteins. The protein concentration was determined using the BCA protein assay kit (Beyotime Biotechnology, Haimen, China). Fifty micrograms of protein samples were electrophoresed and separated by SDS/PAGE (10% gel), and transferred to PVDF membranes (Boster, Wuhan, China). After incubating the membranes for 1 h with blocking buffer at 37°C, blots were incubated with primary antibodies at 4°C for 12 h. Membranes were washed with TBST buffer and incubated with the corresponding secondary antibodies for 1 h at 37°C. ECL kit (Beyotime Biotechnology, Haimen, China) and the gel imaging system (Shanghai Furi Co, Shanghai, China) were used for immune complex detection. GAPDH was used as the loading control. The bands were analyzed by densitometry.

### RT-qPCR

Total RNA was extracted from heart tissue using total RNA extraction kit according to the manufacturer’s instructions. RNA concentrations were determined using optical density measurements at 260 nm on a spectrophotometer. Total RNA was reverse transcribed into cDNA using a cDNA synthesis kit according to the manufacturer’s instructions. Then, 20 μl reaction mixtures containing SYBR green, RNase-free water, a forward primer, a reverse primer, and cDNA were established to detect the relative mRNA expression of the genes according to the manufacturers’ instruction. Primers used in the study are listed in Table 1. The PCR program was performed at 95°C for 10 min, followed by 40 cycles at 60°C for 30 s, 56°C for 30 s, and 72°C for 1 min. Melting curves were used to ensure only the correct product was amplified. Fold change difference compared with sham control group was calculated using **2**^–ΔΔ*C*^_t_ method. Values were normalized against GAPDH values.

**Table 1:** Primer sets for the RT-qPCR amplification

Target gene	Forward primer	Reverse primer
*PPARα*	5′-AACTGACATTTGTGACTG-3′	5′-GTTTCCCATCTCTTGTAAG-3′
*MCAD*	5′-GTCGCGCCAGACTACGATAA-3′	5′-GCCAAGACCACCACAACTCT-3′
*MCPT1*	5′-AAGAACACGAGCCAACAAGC-3′	5’-TACCATACCCAGTGCCATCA-3′
*GAPDH*	5′-GGTGGACCTCATGGCCTACA-3′	5′-CTCTCTTGCTCTCAGTATCCTTGCT-3′

Abbreviation: MCPT1, muscle carnitine palmitoyl transferase-1.

### Statistical analysis

Data are presented as mean ± S.D., and SPSS 13.0 statistical software was used for data processing. Unpaired Student’s *t* test was used for comparison of sham operation group and CHF model group. Comparisons between different doses of AS-IV and the CHF model group were made using ANOVA followed by multiple comparisons using *Dunnett’*’*s* test to evaluate the cardiovascular protective effects of AS-IV, and *P*<0.05 was considered statistically significant.

## Results

### Mortality of the experimental rats

Two rats died in CHF model group and one rat died in CHF + low-dose AS-IV group. No rats died in CHF + Benazepril HCL group, the sham operation group, and the CHF + high-dose AS-IV group. Cause of death may have been associated with heart failure, postoperative infection, or respiratory failure. Prior to AS-IV administration, rats in CHF model group, CHF + low-dose AS-IV group, and CHF + high-dose AS-IV group presented CHF symptoms like tachypnea, loss of appetite, and less activity 3 weeks after the AAC operation. These symptoms were relieved gradually after treatment with AS-IV. The rats in sham operation group did not present CHF symptoms.

### Baseline of the five experimental groups rats before the experiment

To ensure comparability, UCG was conducted in all the rats in the five groups, and there was no difference amongst groups (*P*>0.05) (Table 2).

**Table 2:** The baseline of the five experimental group(s)

Group	*n*	Weeks old (w)	Body weight (g)	LVEDD (mm)	LVESD (mm)	LVPWD (mm)	LVEF (%)
Benazepril HCL	20	8	249 ± 5.5	5.05 ± 0.66	2.39 ± 0.72	1.02 ± 0.10	80.1 ± 3.29
Sham operation	20	8	243 ± 6.1	5.08 ± 0.70	2.41 ± 0.66	0.99 ± 0.12	81.1 ± 3.55
CHF model	20	8	247 ± 5.8	5.06 ± 0.63	2.43 ± 0.65	1.01 ± 0.15	78.9 ± 4.00
Low-dose	20	8	245 ± 5.9	5.03 ± 0.69	2.38 ± 0.68	1.04 ± 0.16	79.6 ± 4.13
High-dose	20	8	250 ± 5.3	5.09 ± 0.65	2.45 ± 0.71	1.03 ± 0.09	80.8 ± 3.60
**LVFS (%)**	**E (m/s)**	**A (m/s)**	**E/A**	**HR (bpm)**	**IVRT (ms)**	**IVRT/HR**	**FFA (mol/l)**
49.10 ± 10.69	0.839 ± 0.07	0.575 ± 0.05	1.459 ± 0.10	331 ± 21	26.31 ± 5.86	0.0795 ± 0.010	322.5 ± 43.1
52.81 ± 9.56	0.855 ± 0.08	0.580 ± 0.06	1.474 ± 0.15	340 ± 26	24.99 ± 5.29	0.0735 ± 0.013	346.7 ± 40.9
50.06 ± 11.51	0.878 ± 0.09	0.583 ± 0.05	1.506 ± 0.12	327 ± 17	25.01 ± 5.51	0.0765 ± 0.015	339.1 ± 45.2
48.09 ± 11.70	0.845 ± 0.06	0.571 ± 0.06	1.480 ± 0.14	339 ± 25	25.95 ± 5.68	0.0766 ± 0.011	350.1 ± 49.2
53.05 ± 12.66	0.871 ± 0.07	0.590 ± 0.04	1.476 ± 0.11	329 ± 19	24.29 ± 5.38	0.0738 ± 0.014	343.5 ± 46.9

Abbreviations: A, mitral valve late diastolic flow propagation velocity; E, mitral valve early diastolic flow propagation velocity; E/A, the ratio of E value to A value; HR, heart rate; IVRT/HR, the ratio of IVRT value to HR value.

### Evaluation of the establishment of the CHF model

**Figure 1:**
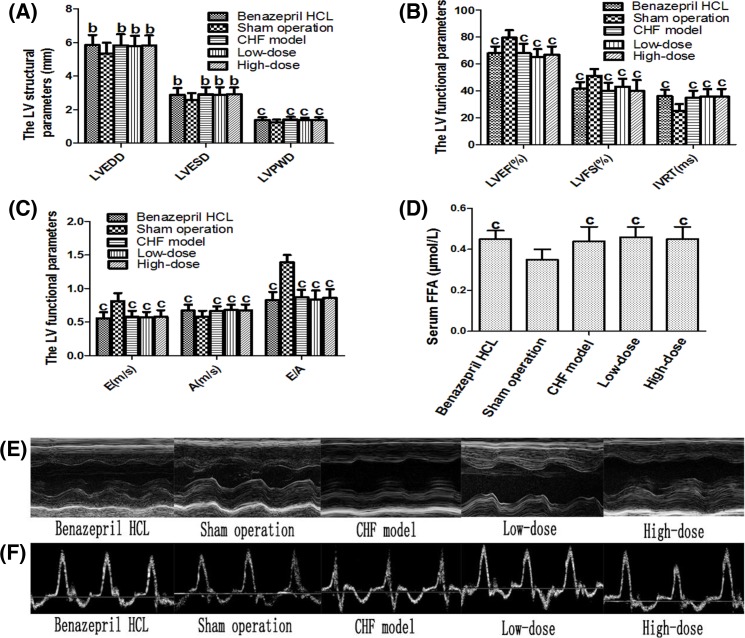
The UCG result of the sham operation rats and the AAC rats (*n*=20 for the CHF + Benazepril HCL group, the sham operation group and the CHF + high-dose group, *n*=18 for the CHF model group and *n*=19 for the CHF+low-dose group) (**A**–**C**) Compared with the sham operation rats, the LVEDD, LVESD, LVPWD, and IVRT of the AAC rats increased, while the LVEF, LVFS, and E/A of the AAC rats decreased. ^b^*P*<0.05 compared with sham operation rats, ^c^*P*<0.01 compared with sham operation rats. (**D**) The serum FFA concentration of the AAC rats increased compared with the sham operation rats. (**E**,**F**) Representative M-mode LV echocardiographs and mitral velocity profiles of the five groups of rats.

Six weeks after AAC operation, UCG was performed for all rats to ensure that CHF model was successfully established. Serum FFA concentrations were also measured. Compared with the sham operation group, rats that underwent AAC operation showed an increase in LVEDD, LVESD, LVPWD, and IVRT and a decrease in LVEF and LVFS indicating that CHF model was successfully established (Figure 1A–C,E,F). Moreover, FFA concentration was higher in AAC operated rats than in sham operated rats. (Figure 1D). There was no statistical difference in these parameters amongst the four groups of rats that received the AAC operation. This basically satisfied the experimental animal CHF standard introduced by Bishop [26].

### Improvement of left ventricular function and structure after AS-IV intervention

**Figure 2:**
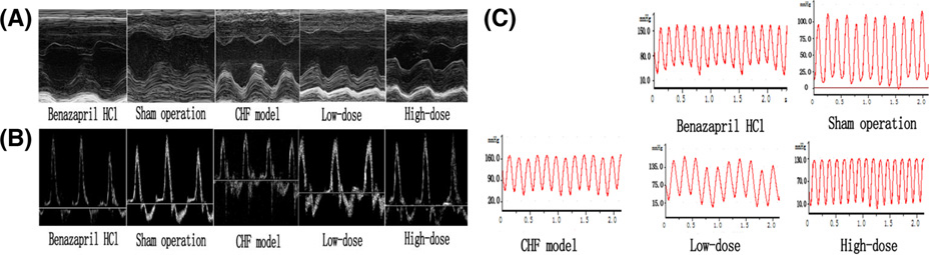
The results after AS-IV intervention. Representative M-mode LV echocardiographs (**A**), mitral velocity profiles (**B**), and left ventricular pressure waveform (**C**) of the five experimental groups after intervention (*n*=20 for the CHF + Benazepril HCL group, the sham operation group, and the CHF + high-dose group, *n*=18 for the CHF model group and *n*=19 for the CHF + low-dose group).

To determine the effects of AS-IV on cardiac function and structure, all five groups underwent UCG and left ventricular intubation after different interventions lasting 8 weeks. From the aspect of cardiac function, the AS-IV treated rats showed an improvement in left ventricular function parameters, including an increased LVEF, LVFS, E/A, and ±d*p*/d*t*_max_ and a decreased IVRT. From the aspect of the left ventricular structure, the AS-IV treated rats showed a decrease in LVEDD, LVESD, and LVPWD compared with the CHF group. Hemodynamically, AS-IV-treated rats showed a decrease in LVEDP and LVESP and an increase in ±d*p*/d*t*_max_ compared with CHF model rats which indicated an improvement in left ventricular function after AS-IV treatment (Tables 3 and 4). This result indicates that AS-IV attenuated cardiac remodeling and improves left ventricular function (Figure 2A–C).

**Table 3:** The UCG result of all of the rats after intervention

Group	*n*	LVEDD (mm)	LVESD (mm)	LVPWD (mm)	LVEF (%)	LVFS (%)
Benazepril HCL	20	6.15 ± 0.61	3.03 ± 0.70	1.62 ± 0.10	67.8 ± 3.89	40.07 ± 5.23
Sham operation	20	6.01 ± 0.99	2.82 ± 0.61	1.59 ± 0.33	79.2 ± 3.55	49.89 ± 6.98
CHF model	18^1^	6.77 ± 0.70^2^	3.49 ± 0.67^3^	1.99 ± 0.26^3^	52.5 ± 5.62^3^	29.01 ± 5.88^3^
Low-dose	19^1^	6.25 ± 0.60^4^	3.08 ± 0.50^4^	1.79 ± 0.19^4^	57.91 ± 6.55^4^	36.56 ± 4.98^5^
High-dose	20	6.19 ± 0.65^4^	3.02 ± 0.71^4^	1.62 ± 0.39^5^	61.8 ± 4.39^5^	41.29 ± 4.56^5^
**E (m/s)**	**A (m/s)**	**E/A**	**HR (bpm)**	**IVRT (ms)**	**IVRT/HR**	
0.659 ± 0.09	0.605 ± 0.07	1.082 ± 0.13	331 ± 26	37.31 ± 5.86	0.1127 ± 0.018	
0.839 ± 0.10	0.566 ± 0.09	1.482 ± 0.16	320 ± 30	26.99 ± 6.06	0.0843 ± 0.015	
0.540 ± 0.06^3^	0.697 ± 0.05^3^	0.775 ± 0.14^3^	397 ± 19^3^	51.01 ± 7.01^3^	0.1284 ± 0.017^3^	
0.597 ± 0.07^4^	0.595 ± 0.04^5^	1.003 ± 0.10^5^	379 ± 26^4^	45.95 ± 4.68^4^	0.1212 ± 0.010	
0.671 ± 0.11^5^	0.583 ± 0.08^5^	1.151 ± 0.12^5^	368 ± 19^5^	33.29 ± 5.38^5^	0.0994 ± 0.015^5^	

Abbreviations: A, mitral valve late diastolic flow propagation velocity; E, mitral valve early diastolic flow propagation velocity; E/A, the ratio of E value to A value; HR, heart rate; IVRT/HR, the ratio of IVRT value to HR value.

^1^Two rats died in the CHF model group, and one rat died in the CHF + low-dose AS-IV group. The quantitative data are shown as the means ± S.D. ^2^*P*<0.05. ^3^*P*<0.01 compared with the sham operation. ^4^*P*<0.05. ^5^*P*<0.01 compared with the CHF model.

**Table 4:** The hemodynamic parameters examined by left ventricular intubation in all the rats after intervention

Group	*n*	Body weight (g)	LVEDP (mmHg)	LVESP (mmHg)	+d*p*/d*t* max (mmHg/s)	−d*p*/d*t* max (mmHg/s)
Benazepril HCL	20	370.1 ± 36.9	16.21 ± 1.95	145.21 ± 4.41	5055 ± 389	5019 ± 489
Sham operation	20	386.5 ± 40.1	5.23 ± 0.82	129.63 ± 4.33	6010 ± 367	6401 ± 609
CHF model	18^1^	360.0 ± 25.3	36.25 ± 1.45^2^	169.86 ± 6.69^2^	4668 ± 359^2^	4023 ± 654^2^
Low-dose	19^1^	375.2 ± 20.1	18.12 ± 1.65^3^	146.15 ± 5.23^3^	4956 ± 454^3^	4401 ± 421^3^
High-dose	20	379.2 ± 30.5	8.82 ± 1.62^3^	120.53 ± 6.01^3^	5339 ± 401^3^	4989 ± 455^3^

The quantitative data are shown as the means ± S.D. Abbreviations: +d*p*/d*t*_max_, LV maximal first derivative of pressure rising over time; –d*p*/d*t*_max_, LV maximal first derivative of pressure dropping over time. ^1^Two rats died in the CHF model group, and one rat died in the CHF + low-dose AS-IV group. ^2^*P*<0.01 compared with the sham operation. ^3^*P*<0.01 compared with the CHF model.

### Gross morphology and LVMI and HMI

**Figure 3:**
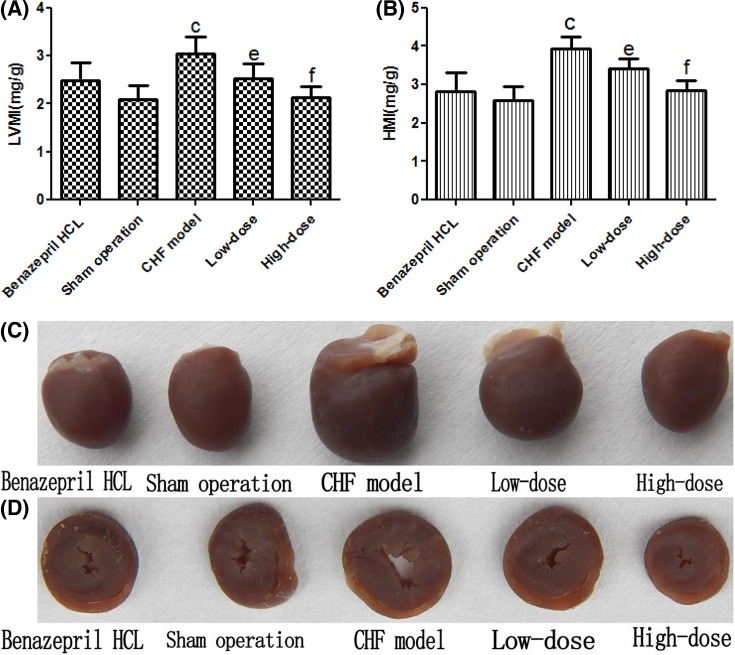
LVMI and HMI and Grossmorphology. The results of the LVMI (**A**) and HMI (**B**) measurement after different interventions (*n*=20 for the CHF + Benazepril HCL group, the sham operation group, and the CHF + high-dose group, *n*=18 for the CHF model group, and *n*=19 for CHF + low-dose group). Representative gross morphology of the whole hearts (**C**) and the corresponding transverse cross-sections at the mid-ventricle level (**D**). ^c^*P*<0.01 comapred with the sham operation, ^e^*P*<0.05 compared with the CHF model, ^f^*P*<0.01 compared with the CHF model.

Rats in all groups were killed painlessly after left ventricular intubation and whole hearts were taken to measure the size and weight of left ventricle and whole heart. Compared with hearts from the sham operation group, cardiac volume of CHF model group was grossly enlarged, and weight of the left ventricle and whole heart also increased (*P*<0.01), which was proven by LVMI and HMI (Figure 3). Compared with hearts from CHF model group, the cardiac volume of CHF + low-dose and CHF + high-dose AS-IV group rats were grossly narrow, and the weight of left ventricle and whole heart was also decreased (*P*<0.05/0.01) (Figure 3).

### Serum and myocardium FFA concentrations

**Figure 4:**
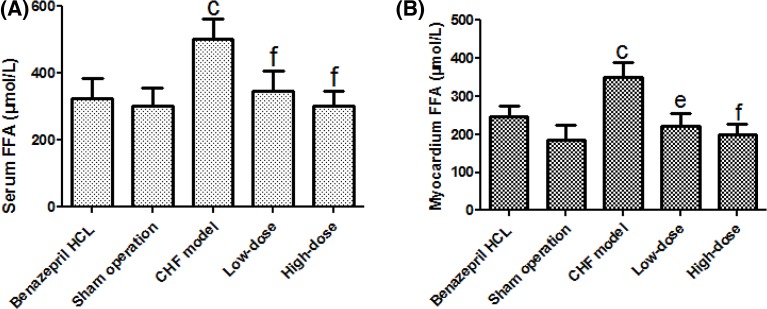
The serum and myocardium FFA concentration. Serum FFA concentration (**A**) and myocardium FFA concentration (**B**) of the five experimental groups. The quantitative data are shown as the means ± S.D. (*n*=20 for the CHF + Benazepril HCL group, the sham operation group, and the CHF + high-dose group, *n*=18 for the CHF model group and *n*=19 for the CHF + low-dose group); ^c^*P*<0.01 compared with the sham operation, ^e^*P*<0.05, ^f^*P*<0.01 compared with the CHF model.

To investigate the effect of AS-IV on FFA metabolism of CHF rats, we determined the serum and myocardium FFA concentrations. The results showed significantly higher levels of serum and myocardium FFA in CHF model group when compared with the sham operation rats (*P*<0.01). In contrast, the serum and myocardium FFA concentration was reduced in AS-IV-treated rats in comparison with CHF model rats (*P*<0.01) (Figure 4). These results indicate that AS-IV improved FFA utilization in CHF rats.

### Histological examination

**Figure 5:**
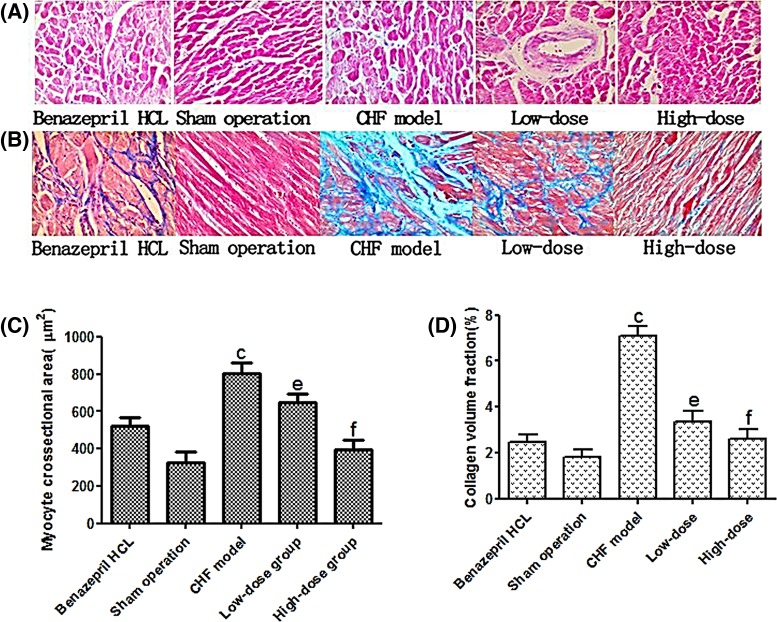
Representative staining sections of the five experimental groups after the interventions (*n*=20 for the CHF + Benazepril HCL group, the sham operation group, and the CHF + high-dose group, *n*=18 for the CHF model group and *n*=19 for the CHF + low-dose group) The sections were visualized at 200× magnification. (**A**) The sections were stained with H&E. (**B**) The sections were stained using Masson’s trichrome. Red indicates the muscle fibers and blue indicates collagen. (**C**) Myocyte cross-sectional area date. (**D**) CVF of the quantitative data. ^c^*P* <0.01 compared with the sham operation, ^e^*P*<0.05, ^f^*P*<0.01 compared with the CHF model.

To determine the histological changes induced by different interventions, we stained the pathological sections using two methods, H&E staining to observe the myocardial cell morphology, and Masson’s trichrome staining to determine the volume of the collagen fibers, which were stained blue (Figure 5A,B).

Compared with the sections in sham operation group, the sections of CHF model group exhibited hypertrophy of the myocardial cells, disorders of cardiomyocyte arrangement, proliferation of collagen fibers, and inflammatory cell infiltration. Treatment with AS-IV reversed these effects in CHF + low-dose and the CHF + high-dose AS-IV group (Figure 5A). The cross-sectional area of myocytes in CHF model rats was higher than that in sham operation rats and AS-IV-treatment reduced this parameter (Figure 5C). Compared with the sections of sham operation group, the CVF of CHF model group sections increased significantly as shown by the Masson’s trichrome staining (*P*<0.01). The CVF of CHF + low-dose and CHF + high-dose AS-IV group sections decreased significantly when compared with CHF group (*P*<0.05/0.01) (Figure 5B,D).

### Western blot examination

**Figure 6:**
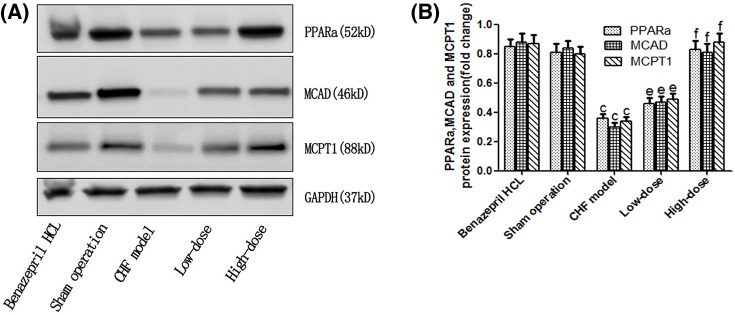
Expression analysis of key enzymes involved in FFA metabolism. Representative blots of PPARα, MCAD, and MCPT1 (**A**) and the intensity normalized against GAPDH (**B**) in the LV myocardium of the five groups (*n*=20 for the CHF + Benazepril HCL group, the sham operation group, and the CHF + high-dose group, *n*=18 for the CHF model group and *n*=19 for the CHF + low-dose group). The quantitative data are given as the means ± S.D. ^c^*P*<0.01 compared with the sham operation; ^e^*P*<0.05, ^f^*P*<0.01 compared with the CHF model.

To investigate the impact of AS-IV on FFA metabolism of myocardial cells, we analyzed the expression of key enzymes involved in FFA metabolism (Figure 6A). Compared with the sham operation group, the protein expression of PPARα, MCAD, and MCPT1 in CHF model group was significantly decreased (*P*<0.01). Importantly, these effects were reversed in CHF + low-dose and the CHF + high-dose AS-IV group rats (*P*<0.05/0.01) (Figure 6B).

### RT-qPCR

**Figure 7:**
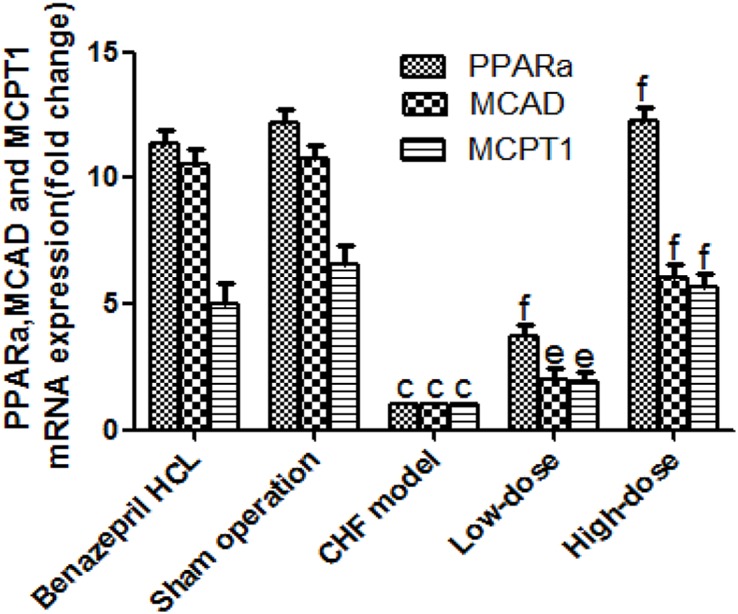
Relative quantitation of *PPARα, MCAD*, and *MCPT1* mRNA by RT-qPCR in the myocardia of the five groups of rats (*n*=20 for CHF + Benazepril HCL group, the sham operation group, and the CHF + high-dose group, *n*=18 for the CHF model group and *n*=19 for the CHF + low-dose group) The quantitative data are given as the means ± S.D. ^c^*P*<0.01 compared with the sham operation; ^e^*P*<0.05, ^f^*P*<0.01 compared with the CHF model. Please note that compared with the sham operation group, CHF + low-dose group was still statistically significant (*P*<0.05); however, the difference in the CHF + high-dose group compared with the sham control group was not significant (*P*>0.05).

We also analyzed the mRNA levels of PPARα, MCAD, and MCPT1 and found that gene expression of these proteins was significantly decreased in CHF model group when compared with the sham operation group (*P*<0.01). Treatment with AS-IV (in CHF + low-dose and CHF + high-dose AS-IV groups) overcame the inhibitory effects of CHF (Figure 7). The high AS-IV dose almost rescued PPARα, MCAD, and MCPT1 expression to the control level (*P*>0.05), while it was only partially rescued in the low-dose group (*P*<0.05 compared with sham control group). These results were consistent with the Western blot data.

## Discussion

In the present study, we demonstrated the cardiovascular protective effect of AS-IV on CHF rats in by measuring four outcomes. Structurally, we proved that AS-IV inhibits ventricular hypertrophy. Functionally, we demonstrated that AS-IV improves left ventricular function. In addition, by gross morphology, we confirmed that AS-IV inhibits ventricular remodeling, proliferation of collagen fibers, and hypertrophy of myocardial cells. The preventive effects of AS-IV on CHF have been proven in many similar studies. Our results are consistent with other studies. Zhang et al. [12] reported that AS-IV protected against cardiac hypertrophy by reducing left intraventricular pressure of rats induced by isoproterenol. In another study, Zhang et al. [27] found that AS-IV improved post-ischemic heart function and ameliorated reperfusion arrhythmias in rat hearts *in vitro*. AS-IV was also found to improve ventricular remodeling in myocardial infarction (MI) and pressure overload (transverse aortic constriction) mouse models [28]. Zhao et al. [17] found that AS-IV attenuated LPS-induced cardiac dysfunction and improved cardiac LVEF, LV end-diastolic volume and the LV end-systolic volume. Zhao et al. [15] reported that AS-IV improved the cardiac functions of congestive heart failure rats induced by ligation of the left coronary artery.

The energy substrates of the heart mainly include FFA and glucose, but in the failing heart, the energy substrates shift from FFA to glucose [29,30]. This is due to the down-regulation of key enzymes in FFA metabolism [31–33]. The key enzymes regulating FFA metabolism include MCPT1 and MCAD, both of which are controlled by the nuclear factor PPARα [34]. MCPT1 is responsible for conversion into fatty acyl carnitine and its uptake by the mitochondria [35]. MCAD regulates the first step in the reaction of fatty acid β-oxidation, namely conversion of acyl-coenzyme A into *trans*-enoyl-CoA [36]. The synthesis of MCPT1 and MCAD is regulated by nuclear factor PPARα the activation of which promotes the expression of downstream genes, namely, the MCPT1 and MCAD [37].

Next, we investigated whether the use of AS-IV will affect the energy substrates of the heart in CHF rats. First, we examined the serum and myocardium FFA concentrations of the different experimental groups. The result showed that the serum FFA concentration of the CHF rats increased obviously compared with the sham operation rats, while the serum and myocardium FFA concentrations in the CHF + AS-IV-treated rats were reduced compared with the CHF model group rats. The consequence is consistent with many other studies, which show that, in the failing heart, the fatty acid concentration often increases [38,39]. Then, we found that the protein of PPARα, MCPT1, and MCAD was increased by Western blot analysis after treatment with AS-IV in contrast with the CHF model rats. This result indicated that AS-IV improves the FFA utilization of the myocardial cell. To confirm this indication, we further detected the mRNA levels of PPARα, MCPT1, and MCAD by RT-qPCR. The data showed that the mRNA levels of PPARα, MCPT1, and MCAD also increased in the CHF + AS-IV-treated rats compared with the CHF model rats. These data are consistent with the Western blot result AVE8134, a PPARα agonist, attenuates the progression of heart failure and increases survival in rats [40]. Fenofibrate, another PPARα agonist, regresses left ventricular hypertrophy and increases the myocardium PPARα expression in spontaneously hypertensive rats [41]. Fenofibrate also inhibits the left ventricle fibrosis of pressure-overload rats induced by abdominal aortic banding [42]. Based on these reports, we rationalized that the activation of PPARα may attenuate the progression of heart failure. In our study, we found that AS-IV inhibited ventricular remodeling, improved the cardiac function, and caused FFA utilization in CHF rats. Our findings suggest that the protective effects of AS-IV on cardiac structure and function are correlated with the activation of PPARα. One potential limitation of the current study is that we have not elucidated the detailed underlying mechanisms by which low- or high-dose of AS-IV causes metabolic reprogramming and how it rescues expression of PPARα, MCAD, and MCPT1 leading to improved FFA utilization. Another limitation of our study is that we did not study the effect of AS-IV on glucose metabolism in CHF model rats. Further studies are needed to understand the relationship between improved lipid utilization and the cardiac function caused by AS-IV.

In summary, our study shows that AS-IV inhibits ventricular remodeling, improves cardiac function, and decreases FFA concentration of CHF model rats. Our findings suggest a therapeutic potential of using AS-IV in CHF.

## Abbreviations

AAC: abdominal aortic constriction
AS-IV: astragaloside IV
Benazepril HCL: benazepril hydrochloride
CHF: chronic heart failure
CMC: carboxymethyl cellulose
CVF: collagen volume fraction
FFA: free fatty acid
GAPDH: glyceraldehyde phosphate dehydrogenase
HMI: heart mass index
IVRT: isovolumic relaxation time
LVEDD: left ventricular end-diastolic dimension
LVEDP: left ventricular end-diastolic pressure
LVEF: left ventricular ejection fraction
LVESD: left ventricular end-systolic dimension
LVESP: left ventricular end-systolic pressure
LVFS: left ventricular fractional shortening
LVMI: left ventricular mass index
LVPWD: left ventricular posterior wall depth
MCAD: medium-chain acyl-CoA dehydrogenase
MCPT1: muscle carnitine palmitoyl transferase-1
PPARα: peroxisome proliferator-activated receptor α
RT-qPCR: real-time quantitative PCR
UCG: ultrasonic cardiogram
